# Association between olfactory identification and cognitive function in community-dwelling elderly: the Shanghai aging study

**DOI:** 10.1186/s12883-016-0725-x

**Published:** 2016-10-20

**Authors:** Xiaoniu Liang, Ding Ding, Qianhua Zhao, Qihao Guo, Jianfeng Luo, Zhen Hong, the Shanghai Aging Study (SAS)

**Affiliations:** 1 Institute of Neurology, Huashan Hospital, Fudan University, WHO Collaborating Center for Research and Training in Neurosciences, Shanghai, 200040 China; 2 Department of Biostatistics, School of Public Health, Fudan University, Shanghai, 200032 China; 3 The Key Laboratory of Public Health Safety, Fudan University, Shanghai, 200032 China; 4 National Clinical Research Center for Aging Diseases, Shanghai, 200040 China

**Keywords:** Olfactory identification, Mild cognitive impairment, Community-based study, Elderly

## Abstract

**Background:**

The smell sense reduction was considered to represent the potentially warning of early stage of neurodegenerative disorders. The Shanghai Aging Study provided us a unique opportunity to explore the association between olfactory identification (OI) and cognitive function among community-dwelling elderly in China.

**Methods:**

OI of each participant was measured by the 12-item identification tests from Sniffin’ Sticks Screening test (SSST-12). Participants with mild cognitive impairment (MCI) were diagnosed by Petersen criteria. We used the logistic regression analysis to explore the association between OI scores and cognitive function by adjusting potential confounders.

**Results:**

Among 1782 non-demented participants, 345 (19.4 %) participants were diagnosed as MCI. The mean OI score for participants with MCI [7.1 (SD 2.3)] was significantly lower than that for those with normal cognition [8.2 (SD 2.0), *P* < 0.0001]. After adjusted for age, gender, education, lifestyles, medical history, Apolipoprotein E genotype, lower OI score was found to be an independent influence factor related to MCI (OR 1.19, 95 % CI 1.11–1.27).

**Conclusions:**

Our study suggests that poor OI may be associated with MCI in elderly population. Further prospective studies may confirm the OI as a reliable and early marker predicting the decline of cognitive function.

## Background

Olfactory function is an important role in health and behavior because it may be a valid marker of the integrity of the aging brain. The prevalence and severity of olfactory dysfunction increase substantially with aging. Olfactory dysfunction represents an important clinical symptom suggestive of early stage of neurodegenerative disorders, including Parkinson’s disease (PD) [1].

A series of community-based and hospital-based studies in western population have demonstrated that in older adults, impaired olfactory function was closely associated with the decline of cognitive functions, especially Alzheimer’s disease (AD) in the preclinical stage, although this dysfunction is more likely to be due to problems of olfactory identification (OI) than detection [2–6]. As a transient condition, mild cognitive impairment (MCI) is gaining more attention. It occurs along the progression from normal aging to dementia, so it comprises a broad clinical spectrum of pre-dementia stages [7]. It was also reported that in MCI, olfactory impairment may herald progression to dementia [8–10].

There was no general standard test for olfactory because of the cultural differences in diverse regions. Modern tests for OI include the Connecticut Chemosensory Clinical Research Center Test (CCCRCT), University of Pennsylvania Smell Identification Test (UPSIT), Cross-Cultural Smell Identification Test (CC-SIT), Pocket Smell Test (PST), Odorant confusion matrix, Biolfa olfactory test, Sniffin’ Sticks (SS), Smell Disketts Test, Scandinavian Odor-identification test (SOIT), and San Diego Odor Identification Test (SDOIT) in western countries [11]. As in Chinese population, only studies in Hongkong have been conducted to investigate the applicability of the OI and threshold tests across the Chinese culture [12, 13].

China has one of the fastest ageing in the world. According to the 2014 census, the number of people aged 60 years and older was 212 million, occupying 15.5 % of the population [14]. So far in mainland China, no large-sampled population-based study was conducted to obtain the epidemiological data related to olfactory function in the elderly, and the previous reports were very limited from just hospital-based studies [12, 15]. The Shanghai Aging Study was a community-based cohort study for investigating the progression of cognitive decline in Chinese elderly [16]. With a study design, operational procedures and diagnostic criteria similar to most cohort studies in developed countries, the Shanghai Aging Study is recognized the first epidemiologic study conducted in mainland China. The Sniffin’ Sticks Smell Test -12 (SSST-12) is a rapid, portable, suited for repetitive and inexpensive screening of OI in a population-based study [17]. By using it, we have a unique opportunity to identify the association between OI and cognitive function in community-dwelling elderly in China.

## Methods

### Study participants

The Shanghai Aging Study was designed to establish a prospective community-based cohort with elderly in downtown Shanghai, China. Eligible participants were registered residents aged 60 or older in Jingansi community, able to communicate and accept physical and cognitive examinations, and they were not suffered from mental retardation or schizophrenia based on their medical records. Recruitment procedures were reported elsewhere [18]. According to the objective of the current study, underlying participants were excluded if they were 1) undergoing maxillofacial surgery, with pathologies of the nose and paranasal sinuses (rhinosinusitis and polyposis, allergic rhinitis); 2) with chronic obstructive pulmonary disease (eg. asthma, chronic sinusitis, etc.), or acute upper respiratory tract infection within 1 week; 3) with dementia or other severe neurological diseases based on their medical record or diagnosed by neurologists; 4) alcohol and drug abuse, which may alter olfaction.

### Clinical interview

Participants were interviewed face-to-face by trained research nurses to obtain information on their demographic characteristics, including age, gender, education, lifestyle factors (such as living alone, cigarette smoking and alcohol drinking). History of chronic diseases, such as physician-diagnosed hypertension, diabetes, and heart disease (including coronary artery disease and arrhythmia), were asked and confirmed from their medical records. Each participant was examined by neurologists for motor responses and reflexes. Neurologists were assigned to administer the Zung Self-Rating Anxiety Scale (ZSAS) and the Center for Epidemiologic Studies Depression Scale (CESD) for each participant to indicate his mood episode within the past week. Anxiety and depression were determined if ZSAS >44 and CESD ≥ 16 [19, 20]. Neurologists also administered the Clinical Dementia Rating (CDR) [21, 22] and Brody Activity of Daily Living (ADL) [23] scale to obtain information on cognitive complaints and activities of daily living, which were used for the diagnosis of cognitive function.

### Neuropsychological assessments

Cognitive function of each participant was tested by a neuropsychological test battery, which covers domains of global cognition, executive function, spatial construction function, memory, language, and attention. The battery contained: 1) Mini Mental State Examination (MMSE); 2) Conflicting Instructions Task (Go/No Go Task); 3) Stick Test; 4) Modified Common Objects Sorting Test; 5) Auditory Verbal Learning Test; 6) Modified Fuld Object Memory Evaluation; 7)Trail-making test A&B; 8) RMB (Chinese currency) test. Normative data and detail description of these tests were reported elsewhere [16, 24]. The neuropsychological tests were administered by study psychometrists according to the education level of each participant. All tests were conducted in Chinese within 90 min.

### Consensus diagnoses for cognitive function

After each clinical and neuropsychological assessment, study neurologists and neuropsychologists (DD, QZ, QG, and ZH) reviewed the functional, medical, neurological, psychiatric, and neuropsychological data and reached a consensus for dementia, MCI and normal cognition. DSM-IV criteria [25] was used to diagnose dementia, while Petersen’s criteria [26] were considered for a diagnosis of MCI. Participants diagnosed with dementia were ineligible to the current study.

### Olfactory identification test

“Sniffin’ Sticks” is a test of nasal chemosensory function based on felt-tip pens that was devised by G. Kobal in Erlangen, Germany. In its most elaborate version, it comprises 3 tests of olfactory function (odor threshold, odor discrimination, and odor identification), and takes approximately 20 to 30 min for application [27]. The SSST-12 is a rapid (approximately 6 min), portable, suited for repetitive and inexpensive screening of OI that utilizes 12 common odors (orange, leather, cinnamon, peppermint, banana, lemon, liquorice, coffee, cloves, pineapple, rose and fish) presented in felt-tip sticks. The SSST-12 has been validated for clinical use in several European countries, such as Germany, United Kingdom, Greece, etc. [17]. In China, only SSST-16 were used in clinical PD studies, however, without validation [28, 29]. In the current study, we used the SSST-12 bought from Burghart Medical Technology [30]. We translated the list of odors into Chinese on the report sheet.

The administrator of OI test was blind for the cognitive diagnosis of each participant. Before the test, participants were reminded to stay away from chewing gum, sweets or cigarettes. Testing was performed in a quiet, air-conditioned room. A brief history including questions related to the participant’s olfactory experience, previous diseases, drug intake, occupation and smoking habits were recorded. The administrator was wearing cotton gloves when presenting the odors. The opened odor sticks were positioned approximately 2 cm in front of both nostrils of each participant. Participants were then asked to sniff for no longer than 3–4 s and to choose one of four answers from a list that described best the odor. An interval of 30 s was between the different sticks.

### APOE genotype assessment

DNA was extracted from blood or saliva, collected from the study participants. Apolipoprotein (APOE) genotyping was conducted by the TaqmanSNP method [31]. The presence of at least one ε4 allele was treated as being APOE-ε4 positive.

### Statistical analysis

The categorical variables were expressed as frequencies (%), and the continuous variables were expressed as the mean and standard deviation (SD). The Pearson Chi-squared test was used to compare the categorical variables. The Student t-test, analysis of variance (ANOVA) and generalized linear model (GLM) were used to compare the continuous variables. We used two multivariate logistic regression models to detect the association between OI and cognitive function, by adjusting for variables of age, gender and education (model 1) and by adjusting additional confounding variables, such as lifestyles (smoking, drinking and living status), medical history (anxiety, depression, heart disease, hypertension and diabetes) and APOE-ε4 allele (model 2). Risk was presented as odds ratio (OR) and 95 % confidence interval (CI). All of the *P*-values and 95 % CIs were estimated in a two-tailed tests. Differences were considered to be statistically significant at *P* < 0.050. The data were analyzed using SAS 9.3 (SAS Institute Inc., Cary, NC, USA).

## Results

### Characteristics of participants

From April 2010 to March 2011, the Shanghai Aging Study consequently enrolled 1808 participants who had completed both of the cognitive assessment and OI test. Twenty-six were excluded due to the diagnosis of dementia. Table 1 showed that, among 1782 participants, 818 (45.9 %) were men. The mean age of the participants was 70.1(SD 7.1) years and mean year of education was 12.3(SD 3.9) years. Three hundred and forty-five (19.4 %) participants were diagnosed as MCI. Characteristics of age, education, MMSE scores, living alone, history of hypertension, diabetes and depression were found to be significantly different between groups with cognitive normal and MCI. The average scores of OI of all the participants were 8.0 (SD 2.1). The average OI scores of participants with MCI [7.1(SD 2.3) scores] were significantly lower than that of participants with normal cognition [8.2 (SD 2.0) scores, *p* < 0.0001].

**Table 1:** Demographic, olfactory identification scores, lifestyles and medical history of the study participants

	All *N* = 1782	Cognitive Normal *N* = 1437	MCI *N* = 345	*P* value
Gender, male, *n* (%)	818(45.9)	659(45.9)	159(46.1)	0.939
Age, years, mean(SD)	70.1(7.1)	69.4(6.8)	73.0(7.8)	<0.0001
Education, years, mean(SD)	12.3(3.9)	12.8(3.5)	10.4(4.9)	<0.0001
MMSE, scores, mean(SD)	28.4(1.8)	28.7(1.5)	27.0(2.5)	<0.0001
OI, scores, mean(SD)	8.0(2.1)	8.2(2.0)	7.1(2.3)	<0.0001
Living alone, *n* (%)	145(8.2)	107(7.5)	38(11.1)	0.035
Cigarette smoking, *n* (%)	185(10.4)	141(9.9)	44(12.8)	0.117
Alcohol drinking, *n* (%)	142(8.0)	114(8.0)	28(8.2)	0.915
Heart disease, * n * (%)	194(10.9)	150(10.5)	44(12.8)	0.223
Hypertension, *n* (%)	962(54.0)	759(52.8)	203(58.8)	0.043
Diabetes, *n* (%)	247(13.9)	180(12.5)	67(19.5)	0.001
Anxiety, *n* (%)	42(2.4)	34(2.4)	8(2.3)	0.969
Depression, *n* (%)	277(15.6)	207(14.4)	70(20.4)	0.008
APOE-ε4 allele positive, *n* (%)	308(18.3)	251(18.4)	57(17.9)	0.837

*P* value is for the comparison between participants with cognitive normal *vs.* MCI

*Abbreviation: SD* standard deviation, *MCI* mild cognitive impairment, *MMSE* Mini-Mental State examination, *APOE* Apolipoprotein, *OI* olfactory identification

### Normative data of olfactory identification scores

Table 2 described the normative data of OI scores in all participants by gender and age groups. In each group with different gender, the mean OI scores decreased by increasing age (P trend < 0.0001). As for the males, the mean OI scores were from 8.4 (SD1.9) in participants aged 60–69 years, to 7.0 (SD 2.3) in participants aged 80 years and above. As for the females, the mean OI scores were from 8.5 (SD1.8) in participants aged 60–69 years, to 6.9 (SD 2.4) in participants aged 80 years and above. There was no significant difference between males and females for the decreasing trend of OI scores by increasing age (*P* = 0.254). The median and percentile scores of OI were also showed in Table 2.

**Table 2:** Description of olfactory identification scores in community-dwelling elderly by gender and age groups

	Male				Female				
Age group, years	60–69	70–79	≥80	*P* value	60–69	70–79	≥80	*P* value	*P* value (male *vs*. female)
No. of participants, *n* (%)	428(50.6)	307(36.3)	83(9.8)		522(49.7)	353 (33.6)	89(8.5)		
Scores, Mean (SD)	8.4(1.9)	7.5(2.1)	7.0(2.3)	<0.0001	8.5(1.8)	7.6(2.0)	6.9(2.4)	<0.0001	0.254
Median (Min, Max)	8(0,12)	8(3,12)	8(0,12)		9(0,12)	8(0,12)	7(0,11)		
Percentile (%)									
10	6	5	4		6	5	4		
20	7	6	5		7	6	5		
30	8	7	6		8	7	6		
40	8	7	7		8	7	7		
50	9	8	7		9	8	7		
60	9	8	8		9	8	8		
70	9	9	8		10	9	8		
80	10	9	9		10	9	9		
90	11	10	9		11	10	10		

*Abbreviation: SD* standard deviation; *P* value is for the comparison of scores of olfactory identification with increasing age

### Association between olfactory identification scores and cognitive function

**Fig. 1:**
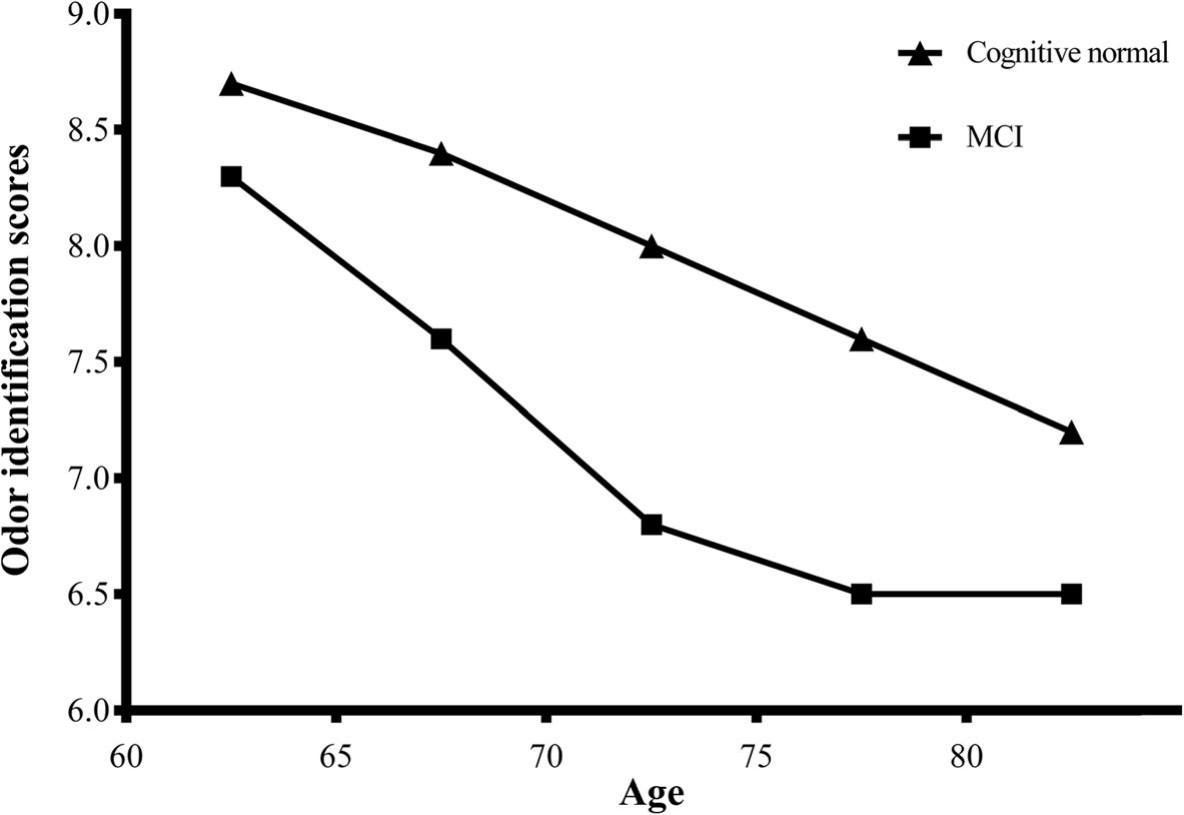
Description of olfactory identification scores in community-dwelling elderly by different cognitions and age groups. *Notes:* Mean OI scores of MCI and cognitive normal groups decreased by increasing age (both *P* trend < 0.0001). The mean OI scores of MCI group decreased more dramatically by increasing age than that of group with normal cognition (*P* < 0.0001). *Abbreviation: OI:* olfactory identification; *MCI:* mild cognitive impairment; *NC:* Normal cognition

**Fig. 2:**
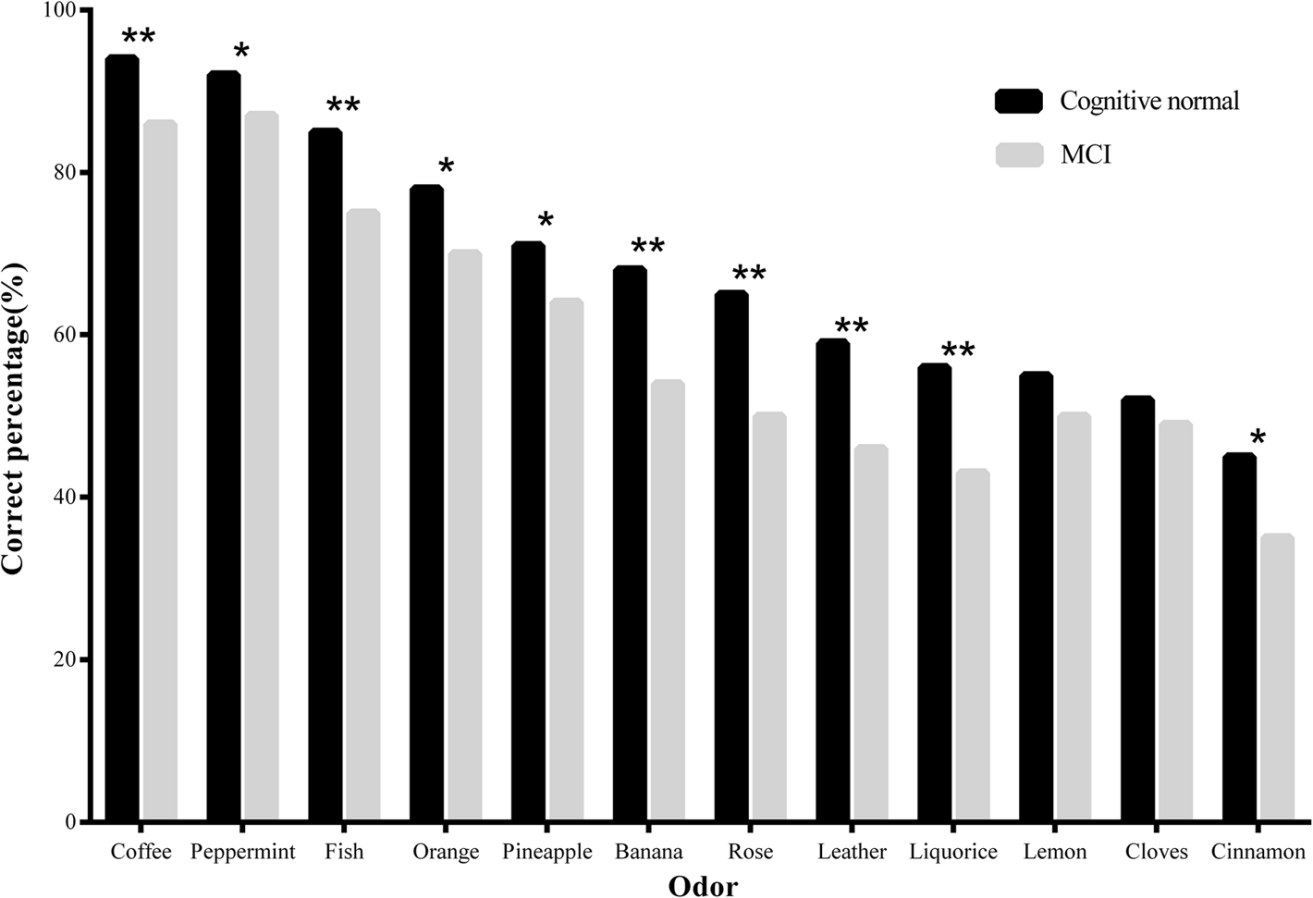
Correct percentages of olfactory identification in community-dwelling elderly with cognition normal and MCI. *Notes:* * represents “*P* < 0.050 and *P* > 0.0001”; ** represents “*P* < 0.0001”

Figure 1 showed that the mean OI scores of MCI and cognitive normal groups decreased by increasing age (both *P* trend < 0.0001). The mean OI scores of MCI group decreased more dramatically by increasing age than that of group with cognitive normal (*P* < 0.0001). Figure 2 demonstrated the correct percentages answered by participants with MCI and cognitive normal for each odor. Three odors with highest correct percentages were coffee (93.6 % for group with cognitive normal, 86.1 % for MCI group), peppermint (91.9 % for group with cognitive normal, 87.3 % for MCI group), and fish (84.6 % for group with cognitive normal, 75.1 % for MCI group). Three odors with lowest correct percentages were lemon (54.8 %), cloves (52.1 %), and cinnamon (45.1 %) for group with cognitive normal, leather (45.8 %), liquorice (43.2 %) and cinnamon (35.4 %) for MCI group. Participants with MCI answered less correct percentages for each odor than those with cognitive normal. Significant differences were found in odor of peppermint (cognitive normal *vs.* MCI, 91.9 % *vs.* 87.3 %%, *P* = 0.015), orange (78.1 % *vs*. 70.4 %, *P* = 0.003), pineapple (70.6 % *vs*. 63.8 %, *P* = 0.009), and cinnamon (45.1 % *vs*. 35.4 %, *P* = 0.001), coffee (93.6 % *vs.* 86.1 %, *P* < 0.0001), fish (84.6 % *vs.* 75.1 %, *P* < 0.0001), banana (68.0 % *vs*. 54.2 %, *P* < 0.0001), rose (65.3 % *vs.* 50.1 %, *P* < 0.0001), leather (58.7 % *vs*. 45.8 %, *P* < 0.0001), and liquorice (56.2 % *vs*. 43.2 %, *P* < 0.0001).

The multivariate logistic model 1 indicated that, lower OI score was an independent influence factor associated with MCI (OR = 1.18, 95 % CI: 1.11–1.25), after adjusted for age, gender and education. Additionally, after adjusting for variables of age, gender, education, living alone, cigarette smoking, alcohol drinking, anxiety, depression, heart disease, hypertension, diabetes, and APOE-ε4 allele, model 2 also indicated that lower OI score was a influence factor independently associated with MCI (OR = 1.19; 95 % CI: 1.11–1.27) (Table 3).

**Table 3:** Odds ratios for olfactory identification score and other confounders among participants with mild cognitive impairment *vs.* cognitive normal

Variable	Model 1 OR (95 % CI)	*P* value	Model 2 OR (95 % CI)	*P* value
OI (score, decreasing)	1.18(1.11,1.25)	<0.0001	1.19(1.11,1.27)	<0.0001
Age (increasing)	1.05(1.03, 1.07)	<0.0001	1.05(1.03, 1.07)	<0.0001
Gender (female *vs.* male)	0.77(0.60, 1.00)	0.053	0.85(0.63, 1.15)	0.284
Education year (increasing)	0.88(0.86, 0.91)	<0.0001	0.88(0.85, 0.91)	<0.0001
Living alone			1.00(0.63, 1.59)	0.995
Cigarette smoking			1.57(0.99, 2.47)	0.053
Alcohol drinking			0.77(0.46, 1.28)	0.309
Heart disease			0.87(0.57, 1.32)	0.506
Hypertension			0.98(0.75, 1.29)	0.896
Diabetes			1.54(1.08, 2.20)	0.018
Anxiety			0.59(0.25, 1.39)	0.227
Depression			1.67(1.18, 2.37)	0.004
APOE-ε4 allele positive			1.04(0.73, 1.46)	0.845

*Model 1:* multivariate logistic regression model, adjusted for age, gender and education

*Model 2:* multivariate logistic regression model, adjusted for age, gender, education, living alone, cigarette smoking, alcohol drinking, anxiety, depression, heart disease, hypertension, diabetes, and Apolipoprotein-ε4

*Abbreviation: OI* olfactory identification, *OR* odds ratio, *CI* confidence interval

## Discussion

To our knowledge, this is the first epidemiological study that reports the normative data of OI among community-dwelling elderly in China by using the SSST-12. The main finding of this community-based study was that, poor OI was an independent factor associated with the cognitive decline in elderly, after adjusting possible confounders. The advantages of this study include community-based design with large sample size, reliable diagnosis of cognitive function based on comprehensive clinical and neuropsychological exams, and detailed influence factor profiles (including APOE genotype) for each participant.

Some small-sampled case-control studies have reported the relation between poor olfaction and AD/MCI. AD patients were more impaired on OI and recognition tasks than on odor detection thresholds task. They were also more strongly impaired on higher-order olfactory tasks involving specific cognitive processes [32]. A study in Germany found that, 7/14 AD patients and 4/8 individuals with MCI showed no olfactory event-related potentials (tested by the SS), suggesting hyposmia, while all comparison individuals had clearly discernible responses [33]. Serby M et al. studied OI in 55 AD patients and 57 controls by using the UPSIT, and found that OI performances were nearly 40 % lower in mild AD patients than in controls [34]. A Hong Kong study revealed that 12 probable AD cases identified significantly fewer odors (tested by Olfactory Identification Test) and had significantly higher olfactory threshold (tested by Alcohol Sniff Test) than their age- and education-matched normal controls [12]. OI (tested by lexical-based OI, lexical-based picture identification, picture-based OI, and odor-detection threshold) was reported to be correlated with cognitive functions in individuals with preclinical AD compared with normal control subjects before they showed any changes in daily functioning [34, 35].

Some prospective studies, mostly in western countries, demonstrated that older persons with impaired OI were more apt to experience cognitive decline than those with relatively preserved OI. The Mayo Clinic Study of Aging followed the 1430 cognitively normal participants over a mean 3.5 years, and observed an association between decreasing olfactory identification with a decreasing number of correct responses in Brief Smell Identification Test (B-SIT) score, and a higher risk of amnestic MCI (aMCI). Compared with the upper B-SIT quartile (quartile [Q] 4, best scores), hazard ratios (HRs) (95 % CI) were 1.12 (0.65–1.92) for Q3 (*P* = 0.680); 1.95 (1.25–3.03) for Q2 (*P* = 0.003); and 2.18 (1.36–3.51) for Q1 (*P* = 0.001) (worst scores; P for trend <0.001) after adjustment for gender and education, with same age. The B-SIT score also predicted progression from aMCI to AD, with a significant dose-response with worsening B-SIT quartiles [6]. The community-based longitudinal study of memory and aging in the Japanese-American community in King County, WA screened 1985 persons using the Cognitive Abilities Screening Instrument (CASI) and the CC-SIT at the baseline. After 2 years, the authors determined an OR for cognitive decline (defined as loss of ≥5.15 points/100 on the CASI) of 0.90 (95%CI: 0.84–0.97) for an increasing in each correct point on the CC-SIT [5]. Another population-based study followed up 1920 older participants in the Epidemiology of Hearing Loss Study in Beaver Dam, Wisconsin. Olfactory was measured by the SDOIT, and the incident cognitive impairment was defined as a MMSE of less than 24 or reported diagnosis of dementia. There was significant association between olfactory impairment at baseline and 5-year incidence of cognitive impairment (OR = 3.72, 95 % CI: 2.31–5.99) after adjusting for possible confounders [2]. The association of OI with decline rate in different cognitive domains was examined in 481 older participants from the Rush Memory and Aging Project. Lower OI score was related to lower function at baseline in each cognitive domain, especially in perceptual speed and episodic memory, after adjustment for age, sex, and education [3]. One hundred seventy-three independent residents of a continuing care retirement community completed UPSIT and MMSE and the Executive Interview (EXIT25) at baseline and were examined twice over 3 years. It concluded that impaired OI non-demented individuals was related to an AD-like memory impairment and an increased rate of cognitive decline [36]. Swan GE et al. reported that, a total of 359 individuals were administered the BSIT for OI, and verbal learning, memory, executive control and global function for cognitive function. Impaired olfactory function was associated with a greater 4.5-year decline on several indices of verbal memory after adjustment for baseline cognitive performance, by a multivariate analysis [37]. Devanand DP et al. examined 90 outpatients with MCI and 45 controls by UPSIT for OI at baseline, and diagnosed incident AD by 2 years. In patients with high MMSE (≥27 of 30), low olfaction with lack of awareness was explored a significant predictor of AD. At follow-up, olfaction scores of 30–35 showed moderate to strong sensitivity and specificity for diagnosis of AD [8].

APOE genotype was identified as an important factor interacting with olfaction. Murphy C et al. have indicated that APOE-ε4 allele is related to OI deficits (tested by SDOIT) in 27 non-demented older persons [38]. Olfactory dysfunction (tested by CC-SIT) in the presence of one or more APOE-ε4 alleles was related to a 4.9 times the risk of cognitive decline in the longitudinal study of memory and aging in the Japanese-American community in King County [5]. A study in China used CC-SIT to assess OI performance in 28 patients with MCI and the 30 controls. They found that individuals with APOE-ε4 allele were able to identify less odors than that of the subjects without APOE-ε4 allele (*P <* 0.010). It suggested that the decreased OI in MCI may be an indicator for the early diagnosis of AD, and APOE genotype may be a portion of the basis of OI decline [15]. Our study did not find the significant contribution of APOE-ε4 allele to cognitive decline through the multivariate logistic regression model. The independent association between the poor OI and cognitive decline were explored, after adjusting the possible confounders including APOE genotype (Table 3).

There were several mechanisms or explanations for the association between olfactory dysfunction and cognition impairment. OI is more strictly involved in cognitive functions since it is processed in the mesial structures of the temporal lobe. The olfactory threshold, however, is mostly influenced by peripheral deficits of the smell sense. It has been shown that typical lesions of AD (i.e., amyloid core and neurofibrillar tangles) were already detectable in the preclinical stages in central olfactory pathways [39]. With recent reports in pathological and brain imaging analyses, it has been found that neurodegenerative changes, including the appearance of Lewy bodies, were commonly observed in regions of the brain which responsible for olfactory perception, such as the amygdala, hippocampus, and orbitofrontal cortex. These changes are apparent from the earliest stages of dementia [40, 41]. Kjelvik G et al studied 12 patients with aMCI, 6 with early AD, and 30 controls. They reported that the aMCI/AD group with reduced OI ability (measured by B-SIT and SSIT), had significantly smaller hippocampal volume than that of the patient group with OI scores > 50 %. There was a significant association between OI scores and hippocampal volume only in the patient group. The results suggested that the decrease in the hippocampus size in connection with early AD was related to more with loss of OI ability rather than loss of memory, thus demonstrating that impaired OI is an early indicator of medial temporal lobe degeneration [42].

Our study also explored an interesting finding that, some odors, such as coffee, fish, banana, rose, leather, and liquorice were identified more significantly different between individuals with MCI and cognitive normal, than odors of lemon and cloves. It suggests that some odors might be the sensitive indicators for the early stage of cognitive decline, while some might be not. The transformational mechanism of stimulation by different odors may need more attention.

Olfactory function includes threshold, discrimination, identification and olfactory recognition [11]. In the current study, we only studied the OI in our study participants. It was reported that odor discrimination and identification performance correlated more significantly than detection thresholds with performance on neuropsychological tests [9]. Thus other domains of olfaction were also important, especially discrimination, which could also be useful as the early predictor of AD. The second limitation is the lack of assessment for parkinsonism and PD, because the olfactory dysfunction occurs in early stages of clinical PD and in asymptomatic relative of PD patient with a prevalence of approximately 90 % [43]. Other limitations include the cross-sectional study design, which cannot determine the causal association, so it is possible that the poor OI was the early symptom of MCI rather than a risk factor; and the potentially unmeasured confounding factors, which may influence the results.

## Conclusion

Our study suggests that poor OI may be associated with MCI in elderly population. Follow-up assessment of our Shanghai Aging Study cohort will be carried out to confirm olfactory dysfunction may be an important predictor for cognitive impairment, as well as its pathological mechanism.

## Abbreviations

AD: Alzheimer’s disease
ADL: Brody activity of daily living
aMCI: Amnestic MCI
ANOVA: Analysis of variance
APOE: Apolipoprotein
B-SIT: Brief smell identification test
CASI: Cognitive abilities screening instrument
CCCRCT: Connecticut Chemosensory Clinical Research Center Test
CC-SIT: Cross-cultural smell identification test
CDR: Clinical dementia rating
CESD: Epidemiologic studies depression scale
CI: Confidence interval
EXIT25: Executive interview
GLM: Generalized linear model
HRs: Hazard ratios
MCI: Mild cognitive impairment
MMSE: Mini mental state examination
NC: Normal cognition
OI: Olfactory identification
OR: Odds ratio
PD: Parkinson’s disease
PST: Pocket smell test
SD: Standard deviation
SDOIT: San Diego odor identification test
SOIT: Scandinavian odor-identification test
SS: Sniffin’ sticks
SSST: Sniffin’ sticks screening test
UPSIT: University of Pennsylvania smell identification test
ZSAS: Zung self-rating anxiety scale

## References

[1] Attems J, Walker L, Jellinger KA (2015). Olfaction and aging: a mini-review. Gerontology.

[2] Schubert CR, Carmichael LL, Murphy C, Klein BE, Klein R, Cruickshanks KJ (2008). Olfaction and the 5-year incidence of cognitive impairment in an epidemiological study of older adults. J Am GeriatrSoc.

[3] Wilson RS, Arnold SE, Tang Y, Bennett DA (2006). Odor identification and decline in different cognitive domains in old age. Neuroepidemiology.

[4] Sohrabi HR, Bates KA, Rodrigues M, Taddei K, Laws SM, Lautenschlager NT, Dhaliwal SS, Johnston AN, MacKay-Sim A, Gandy S, Foster JK, Martins RN (2009). Olfactory dysfunction is associated with subjective memory complaints in community-dwelling elderly individuals. J Alzheimers Dis.

[5] Graves AB, Bowen JD, Rajaram L, McCormick WC, Mccurry SM, Schellenberg GD, Larson EB (1999). Impaired olfaction as a marker for cognitive decline: interaction with apolipoprotein e epsilon4 status. Neurology.

[6] Roberts RO, Christianson TJ, Kremers WK, Mielke MM, Machulda MM, Vassilaki M, Alhurani RE, Geda YE, Knopman DS, Petersen RC. Association between olfactory dysfunction and amnestic mild cognitive impairment and Alzheimer disease dementia. JAMA Neurol. 1-9. doi: 10.1001/jamaneurol.2015.2952. [Epub ahead of print] 10.1001/jamaneurol.2015.2952 PMC4710557 26569387

[7] Petersen RC, Doody R, Kurz A, Mohs RC, Morris JC, Rabins PV, Ritchie K, Rossor M, Thal L, Winblad B (2001). Current concepts in mild cognitive impairment. Arch Neurol.

[8] Devanand DP, Micheals-Marston KS, Liu X, Pelton GH, Padilla M, Marder K, Bell K, Stern Y, Mayeux R (2000). Olfactory deficits in patients with mild cognitive impairment predict Alzheimer’s disease at follow-up. Am J Psychiatry.

[9] Djordjevic J, Jones-Gotman M, De Sousa K, Chertkow H (2008). Olfactory in patients with mild cognitive impairment and Alzheimer’s disease. Neurology Aging.

[10] Yoon JH, Kim M, Moon SY, Yong SW, Hong JM (2015). Olfactory function and neuropsychological profile to differentiate dementia with lewy bodies from Alzheimer’s disease in patients with mild cognitive impairment: a 5-year follow-up study. J Neurologial Sciences.

[11] Eibenstein A, Fioretti AB, Lena C, Rosati N, Amabile G, Fusetti M (2005). Modern psychophysical tests to assess olfactory function. NeurolSci.

[12] Chan A, Tam J, Murphy C, Chiu H, Lam L (2002). Utility of olfactory identification test for diagnosing Chinese patients with alzheimer’s disease. J ClinExpNeuropsychol.

[13] Liu HC, Wang SJ, Lin KP, Lin KN, Fuh JL, Teng EL (1995). Performance on a smell screening test (the MODSIT): a study of 510 predominantly illiterate Chinese subjects. Physiol Behav.

[14] Population of over-60-yr-olds reaches 212 million. 2015. Available at: http://www.newsgd.com/news/2015-06/15/content_126401545.htm. Accessed 13 Jan 2016.

[15] Wang QS, Tian L, Huang YL, Qin S, He LQ, Zhou JN (2002). Olfactory identification and apolipoprotein e epsilon 4 allele in mild cognitive impairment. Brain Res.

[16] Ding D, Zhao Q, Guo Q, Meng H, Wang B, Luo J, Mortimer JA, Borenstein AR, Hong Z (2015). Prevalence of mild cognitive impairment in an urban community in China: a cross-sectional analysis of the Shanghai aging study. Alzheimers Dement.

[17] Hummel T, Konnerth CG, Rosenheim K, Kobal G (2001). Screening of olfactory function with a four-minute odor identification test: reliability, normative data, and investigations in patients with olfactory loss. Ann OtolRhinolLaryngol.

[18] Ding D, Zhao Q, Guo Q, Meng H, Wang B, Yu P, Luo J, Zhou Y, Yu L, Zheng L, Chu S, Mortimer JA, Borenstein AR, Hong Z (2014). The Shanghai aging study: study design, baseline characteristics, and prevalence of dementia. Neuroepidemiology.

[19] Zung WW (1971). A rating instrument for anxiety disorders. Psychosomatics.

[20] Zhang J, Norvilitis JM (2002). Measuring chinese psychological well-being with western developed instruments. J Pers Assess.

[21] Morris JC (1993). The clinical dementia rating (cdr). current version and scoring rules. Neurology.

[22] Lim WS, Chong MS, Sahadevan S (2007). Utility of the clinical dementia rating in asian populations. Clin Med Res.

[23] Lawton MP, Brody EM (1969). Assessment of older people: self-maintaining and instrumental activities of daily living. Gerontologist.

[24] Zhang MY, Katzman R, Salmon D, Jin H, Cai GJ, Wang ZY, Qu GY, Grant I, Yu E, Levy P, Klauber MR, Liu WT (1990). The prevalence of dementia and alzheimers-disease in shanghai, china - impact of age, gender, and education. Ann Neurol.

[25] American Psychiatric Association (1994). Diagnostic and statistical manual of mental disorders.

[26] Petersen RC (2004). Mild cognitive impairment as a diagnostic entity. J Intern Med.

[27] Kobal G, Klimek L, Wolfensberger M, Gudziol H, Temmel A, Owen CM, Seeber H, Pauli E, Hummel T (2000). Multicenter investigation of 1,036 subjects using a standardized method for the assessment of olfactory function combining tests of odor identification, odor discrimination, and olfactory thresholds. Eur Arch Otorhinolaryngol.

[28] Chen W, Chen S, Kang WY, Li B, Xu ZM, Xiao Q, Liu J, Wang Y, Wang G, Chen SD (2012). Application of odor identification test in Parkinson’s disease in china: a matched case-control study. J NeurolSci.

[29] Chen W, Tan YY, Hu YY, Zhan WW, Wu L, Lou Y, Wang X, Zhou Y, Huang P, Gao Y, Xiao Q, Chen SD (2012). Combination of olfactory test and substantia nigra transcranial sonopraphy in the differential diagnosis of Parkinson’s disease: a pilot study from china. TranslNeurodegener.

[30] TinsdalerWeg 175, Burghart Medical Technology, Hamburg, Germany. http://www.burghart.net. Accessed 13 Jan 2016.

[31] Smirnov DA, Morley M, Shin E, Spielman RS, Cheung VG (2009). Genetic analysis of radiation-induced changes in human gene expression. Nature.

[32] Rahayel S, Frasnelli J, Joubert S (2012). The effect of alzheimer’s disease and parkinson’s disease on olfaction: a meta-analysis. Behav Brain Res.

[33] Peters JM, Hummel T, Kratzsch T, Lotsch J, Skarke C, Frolich L (2003). Olfactory function in mild cognitive impairment and alzheimer’s disease: an investigation using psychophysical and electrophysiological techniques. Am J Psychiatry.

[34] Serby M, Larson P, Kalkstein D (1991). The nature and course of olfactory deficits in alzheimers-disease. Am J Psychiat.

[35] Morgan CD, Nordin S, Murphy C (1995). Odor identification as an early marker for alzheimers-disease - impact of lexical functioning and detection sensitivity. J ClinExpNeuropsyc.

[36] Royall DR, Chiodo LK, Polk MJ, Jaramillo CJ (2002). Severe dysosmia is specifically associated with Alzheimer-like memory deficits in nondemented elderly retirees. Neuroepidemiology.

[37] Swan GE, Carmelli D (2002). Impaired olfaction predicts cognitive decline in nondemented older adults. Neuroepidemiology.

[38] Murphy C, Bacon AW, Bondi MW, Salmon DP (1998). Apolipoprotein e status is associated with odor identification deficits in nondemented older persons. Ann N Y AcadSci.

[39] Morris JC, Storandt M, Miller JP, Mckeel DW, Price JL, Rubin EH, Berg L (2001). Mild cognitive impairment represents early-stage alzheimer disease. Arch Neurol.

[40] Hubbard PS, Esiri MM, Reading M, Mcshane R, Nagy Z (2007). Alpha-synuclein pathology in the olfactory pathways of dementia patients. J Anat.

[41] Silveira-Moriyama L, Holton JL, Kingsbury A, Ayling H, Petrie A, Sterlacci W, Poewe W, Maier H, Lees AJ, Revesz T (2009). Regional differences in the severity of lewy body pathology across the olfactory cortex. NeurosciLett.

[42] Kjelvik G, Saltvedt I, White LR, Stenumgard P, Sletvold O, Engedal K, Skatun K, Lyngvaer AK, Steffenach HA, Haberg AK (2014). The brain structural and cognitive basis of odor identification deficits in mild cognitive impairment and alzheimer’s disease. BmcNeurol.

[43] Doty RL, Stern MB, Pfeiffer C, Gollomp SM, Hurtig HI (1992). Bilateral olfactory dysfunction in early stage treated and untreated idiopathic Parkinson’s disease. J Neurol Neurosurg Psychiatry.

